# The incidence of severe oral mucositis and its occurrence sites in pediatric oncologic patients

**DOI:** 10.4317/medoral.24185

**Published:** 2020-11-28

**Authors:** Juliana Ramalho Guimarães, Lais Guedes Alcoforado de Carvalho, Lecidamia Cristina Leite Damascena, Maria Eduarda Alves Sampaio, Isabella Lima Arrais Ribeiro, Simone Alves de Sousa, Ana Maria Gondim Valença

**Affiliations:** 1 Ph.D. student in Dental Sciences, Graduate Program in Dentistry, Federal University of Paraíba, João Pessoa, Brazil; 2 Ph.D. student in Health Decision Models, Federal University of Paraíba, João Pessoa, Brazil; 3 M.Sc. in Dental Sciences, Graduate Program in Dentistry, Federal University of Paraíba, João Pessoa, Brazil; 4 Ph.D. in Health Decision Models, Federal University of Paraíba, João Pessoa, Brazil; 5 Professor at the Department of Clinics and Social Dentistry, Federal University of Paraíba, João Pessoa, Brazil; 6 Professor at the Graduate Program in Health Decision Models, Federal University of Paraíba, João Pessoa, Brazil

## Abstract

**Background:**

Childhood cancer is one of the main causes of child mortality and its treatment has debilitating effects on the oral cavity. Several oral mucositis (SOM) is one of the most common and may cause undesirable symptoms such as pain and risk of systemic infection.

**Material and Methods:**

This was a longitudinal, retrospective, and observational study determining the incidence of severe oral mucositis (SOM) and its occurrence sites in pediatric oncologic patients, in João Pessoa, Brazil, between 2013 and 2018. Data from 56 patients aged 1 to 18 years were collected from their medical records and through an oral mucosa examination, from the 1st to 5th week of chemotherapy treatment (CT) using the modified Oral Assessment Guide, by previously calibrated examiners (Kappa index > 0.7). The data were analyzed by the Chi-square test, and Odds Ratios were calculated.

**Results:**

Most patients were females (54.5%), aged 8.8 years (± 4.8), with hematologic tumors (73.2%), predominantly Acute Lymphoid Leukemia (50.0%). An increase in the occurrence of SOM was observed throughout the CT (*P* = 0.05), ranging from 12.5% in the 1st to 35.7% in the 5th CT week. In the 1st CT week, there was a predominance of alterations in the lips (5.5%) and saliva (5.5%), while in the 5th, the jugal / palate mucosa (21.4%) remained the most affected site by SOM. Differences in the severity of SOM in the jugal / palate mucosa (*P* = 0.01) and labial mucosa (*P* = 0.04) were observed over time. In the 5th CT week, the likelihood of developing SOM was 13.3-fold higher (95% CI: 1.5 - 105.6) in patients with hematologic tumors.

**Conclusions:**

The incidence of SOM was higher in the 5th CT week, most commonly affecting the jugal / palate mucosa, and patients with hematologic tumors were more prone to develop SOM.

** Key words:**Mucositis, oncology, pediatric dentistry.

## Introduction

Childhood cancer is the second leading cause of child mortality worldwide (1). Several protocols have been currently used in cancer treatment, although some of which have debilitating side effects on the oral cavity, such as oral mucositis (2). Clinically, mucositis presents as painful, ulcerated, erythematous areas, which cause intense discomfort, pain, and dysphagia (3).

Oral mucositis may manifest to varying degrees of severity: mild, moderate, and severe. Severe oral mucositis (SOM) is highly prevalent, painful, and poses an increased risk for systemic infection, thus reducing patient survival (2,4,5).

SOM is a multifactorial condition that seems to depend extensively on variables related to individual aspects. Factors such as race, sex, age, genetic factors, tumor type, oral hygiene, treatment length and relapse might be contributing factors. Although is not clarified yet the variables more associated to SOM (4,6,7). Therefore, the clinical characteristics, development course and duration of SOM warrant further investigation (8).

Analyzing the factors that contribute to the progression of SOM in children undergoing chemotherapy (CT) could guide the healthcare team through palliative care and may determine the success of antineoplastic treatment. Thus, the present study aimed to determine the incidence of SOM and its occurrence sites in pediatric oncologic patients admitted to a referral hospital.

## Material and Methods

This was a longitudinal, retrospective, and observational study analyzing medical records and the oral mucosa of patients undergoing antineoplastic treatment from 2013 to 2018 at the Napoleão Laureano Hospital (HNL), located in the city of João Pessoa, Paraíba, Brazil. This philanthropic hospital is considered a referral center for cancer treatment. The study was previously approved by the Research Ethics Committee of the Federal University of Paraíba, under protocol number 64249317.3.0000.5188.

The study sample consisted of 56 eligible individuals selected by convenience sampling. Patients aged 1 to 18 years, undergoing chemotherapy, who developed SOM, were considered eligible. Those who did not undergo chemotherapy, nor were followed up for five consecutive weeks, were excluded from the analysis.

The data were collected during weekly evaluations with 7-day intervals in between them for five consecutive weeks. The patients included in our study had their oral mucosa examined using the modified Oral Assessment Guide (OAG) criteria proposed by Cheng, Chang and Yuen (9). The modified OAG was applied by previously calibrated examiners (Kappa index > 0.7).

The OAG is an easy-to-apply instrument which considers eight items, namely: voice, swallowing, lips, tongue, saliva, labial / palate mucosa, labial mucosa, and gingiva. Each anatomical site is assigned a value (1 to 3), where #1 represents normality, #2 means moderate alteration, and #3 indicates the establishment of SOM. If any of the eight items met the Stage 3 requirements, then the patient was diagnosed with SOM.

Chi-square test was used to check for an association between the occurrence of SOM and the items / sites evaluated by the modified OAG, as well as to identify differences in the occurrence of SOM related to tumor type (hematologic vs. solid) throughout the follow-up period. Odds Ratios (OR) and 95% confidence intervals (CI) were used to calculate the risk estimates of children and adolescents with cancer developing SOM, considering the tumor type and the CT week.

The data were analyzed in SPSS version 20.0, considering a 5% significance level.

## Results

The study sample consisted of 56 patients, most of them were females (n = 31; 55.4%), with an average age of 8.8 years (± 4.8). There was a higher incidence of hematologic cancer in our study sample (73.2%) than in solid tumors (26,8%), with a predominance of acute lymphoid leukemia (50.0%) (Table 1). Other types of cancer were identified in the sample and are shown in Table 1.

**Table 1:**
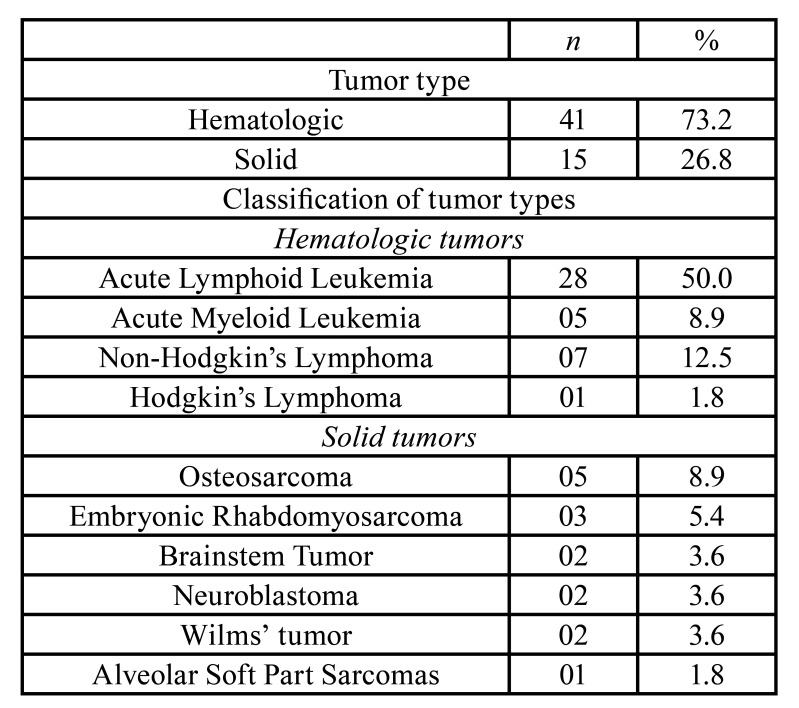
Characterization of patients included in study.

SOM developed in the first week of CT treatment with an increase in the occurrence of SOM over the CT sessions, ranging from 12.5% in the 1st CT week to 35.7% in the 5th CT week (*P* = 0.05) (Table 2).

In the 1st CT week, there was a predominance of alterations in the lips (5.5%) and saliva (5.5%). In the 2nd week, the labial mucosa (19.6%) was the most affected site. In the 3rd week, the lips (16.1%) and jugal / palate mucosa (17.9%) were the sites most commonly affected by SOM. In the 4th week, there was a higher frequency of alterations in the labial mucosa (8.9%) and jugal / palate mucosa (10.7%), while in the 5th week, the jugal / palate mucosa (21.4%) remained the most affected site by SOM (Table 3).

Throughout the CT weeks, anatomical sites in the oral cavity showed different susceptibility to SOM. The jugal / palate mucosa (P-value = 0.010) and the labial mucosa (P-value = 0.037) were the sites most commonly compromised as compared to others in the oral cavity (Table 3).

Pediatric patients with hematologic tumors were 13.33 fold more likely (95% CI: 1.6 - 110.9) to develop SOM than those diagnosed with solid tumors (Table 4).

**Table 2:**
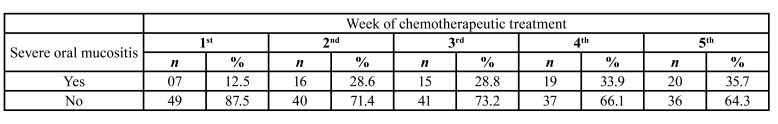
Variability in the incidence of severe oral mucositis throughout the chemotherapy treatment (1st to 5th week) of oncologic pediatric patients.

**Table 3:**
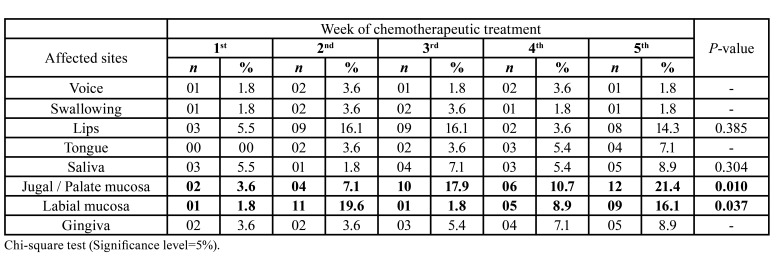
Association between the occurrence of severe oral mucositis and the week of chemotherapeutic treatment in each site of occurrence according to modified Oral Assessment Guide.

**Table 4:**
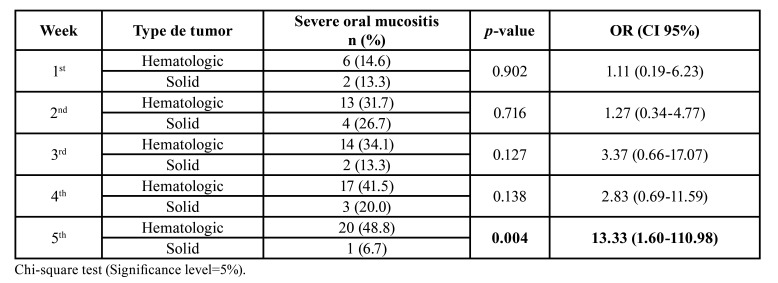
Association between tumor type (hematologic or solid) and the occurrence of severe oral mucositis during chemotherapy.

## Discussion

Our study conducted an evaluation of pediatric oncologic patients from 1 to 18 years old for a follow-up period of 5 weeks. There was variation in SOM incidence during the CT week and it was higher in the 5th CT week. We observed changes in the components of the stomatognathic system according to the modified OAG for a follow-up period of 5 weeks.

The findings of our study showed that oral changes caused by oral mucositis were present at 1st CT week. This fact could be explaining because the complex pathophysiological processes of chemoradiotherapy induced OM starts immediately after the administration of the cytotoxic agents (10,11). The early recognition of clinical signs of oral mucositis and, especially, the anatomical sites commonly affected by chemoradiotherapy-induced OM is essential to prevent worsening of the condition. This approach contributes, for example, to do not be necessary reduction and even cessation of cancer treatment (11,12).

Concerning the sex of the patients, our findings differ from the literature, which reports a higher incidence of childhood malignancies in male individuals (1,4,13). This may be explained by the fact that the city of João Pessoa tends to show a slightly higher incidence rate of childhood leukemia in the female population, according to a study conducted in different regions of Brazil (14).

The mean age of the patients was similar to that reported elsewhere (4,15). Age is a contributing factor to the onset of oral mucositis. The CT effects on the oral mucosa appear to be more frequent in younger patients due to their higher mitotic activity (16,17).

The higher incidence of hematologic neoplasms, observed in our study sample, was also reported on literature (4,7,18). Among the hematologic malignancies, there was a predominance of acute lymphoid leukemia (50.0%). This type of cancer is more common in early childhood especially in boys aged 1 to 4 years (14,19).

SOM developed in the first week of CT treatment. Oral mucositis can manifest as early as three days after the start of CT until 28 weeks thereafter, as reported in a study with 104 pediatric patients undergoing CT (5).

Our findings indicated an increase in the occurrence of SOM over the CT sessions. Villa and Sonis (20) reported that chemo-induced oral mucositis may occur between the second and third week of treatment.

Consistent with our findings, studies have shown that the non-keratinized mucosa of the soft palate, jugal mucosa, lateral tongue and lips are considered sites more prone to develop oral mucositis (21,22).

The modified OAG also indicated salivary changes in patients undergoing CT. Another consequence is the reduction in saliva production, which causes the sensation of xerostomia or dry mouth (3). This is a result of the deleterious effects of antineoplastic therapy on salivary glands predisposing oncologic patients to reduced salivary flow (23).

Considering the importance of recognizing the occurrence of changes in the components of the stomatognathic system of patients receiving conventional chemotherapy, this study adopted the modified OAG (9). This instrument considers oral conditions that are not commonly addressed in other assessment tools (6) and allows the weekly monitoring of the oral health conditions in pediatric cancer patients (12).

Throughout the chemotherapeutic treatment weeks, anatomical sites in the oral cavity showed different susceptibility to SOM. The jugal / palate mucosa and the labial mucosa were the sites most commonly compromised. A cross-sectional study with children and adolescents undergoing CT observed that the tongue and jugal mucosa areas were most frequently compromised by SOM (24).

Oral complications are more frequent in patients with hematologic malignancies (18). A study conducted in India identified a higher incidence of oral mucositis in those with hematologic cancer (15). Individuals with acute leukemia are at higher risk of developing oral mucositis, because they have high concentrations of pro and anti-inflammatory cytokines and low levels of pro-LL-37 in plasma, a protein that was shown to have a protective role in oral health. This suggests that these clinical parameters influence the onset and progression of oral mucositis in this group (5).

An appropriate oral hygiene routine can mitigate the severity of oral mucositis, given that imbalances in biofilm composition due to poor oral hygiene could exacerbate the inflammatory process (25). Moreover, thriving pathogenic biofilms can significantly disturb the host epithelial wound healing capacity (26). Therefore, preventive strategies (e.g., educational programs) are recommended and should be implemented (27).

Based on a longitudinal study design, our findings provide an overview of how oral mucositis develops throughout the CT treatment. The anatomical sites mainly affected by oral mucositis in oncologic pediatric patients over consecutive CT cycles were identified. Special attention should be drawn to these areas in order to mitigate the severity of the condition.

Childhood cancer has a low prevalence in the population, which explains the reduced sample size of our study. This limitation was also reported by other authors, who pointed out that single-center cancer studies can hardly recruit enough eligible patients to perform randomized controlled clinical trials (9). An important strength of our study consists in its longitudinal design. Most studies in this field have a cross-sectional design and provided limited quality of evidence (28) and only a few studies have addressed the pediatric oncologic population.

Moreover, there are other explanatory variables that presumably affect the occurrence of SOM, such as oral hygiene condition, the cytotoxic agents used and the dose administered which are not evaluated in this study.

Our findings may guide the implementation of oral care strategies for pediatric patients receiving chemotherapeutic agents by indicating the sites most likely affected by a severe form of mucositis. A thorough evaluation and early identification of changes resulting from chemotherapy should be performed. With this, the follow-up and management of pediatric oncologic patients susceptible to oral mucositis are rendered more effective.

In this study, severe oral mucositis manifested since the first week of chemotherapeutic treatment and had its incidence increased over time. The fifth week of treatment had the highest occurrence of severe oral mucositis, which was detected in typical oral sites. Pediatric oncologic patients with hematologic tumors were more likely to develop severe oral mucositis than those with solid tumors.
